# ANO1 (TMEM16A) Genetic Variants, Promoter Methylation, and Chloride Dysregulation in Pulmonary Hypertension

**DOI:** 10.3390/jcdd13060283

**Published:** 2026-06-22

**Authors:** İrfan Yaman, Hasan Korkmaz, Arzu Etem Akağaç, Tuğçe Kaymaz, Rauf Önder, Ebru Etem Önalan

**Affiliations:** 1 Department of Cardiology, Faculty of Medicine, Firat University, 23119 Elazig, Turkey; hkorkmaz@firat.edu.tr (H.K.); ebruetem@firat.edu.tr (E.E.Ö.); 2 Department of Biochemistry, Uskudar State Hospital, 34668 Istanbul, Turkey; arzuetem@yahoo.com; 3 Department of Molecular Biology and Genetics, Nevsehir Haci Bektas Veli University, 50300 Nevsehir, Turkey; tugcekaymaz@nevsehir.edu.tr; 4 Department of Cardiology, Gaziantep City Hospital, 27470 Gaziantep, Turkey; drraufonder@gmail.com

**Keywords:** pulmonary arterial hypertension, ANO1, rs7127129, rs2509153, methylation, chloride channels

## Abstract

Background: Pulmonary arterial hypertension (PAH) is a rare and progressive disorder characterized by increased pulmonary vascular resistance and vascular remodeling. Genetic polymorphisms, epigenetic modifications, and ion channel dysregulation are increasingly recognized as key contributors to disease pathogenesis. Anoctamin-1 (ANO1/TMEM16A), a calcium-activated chloride channel, plays a critical role in vascular tone regulation. Objective: This study aimed to investigate the association between ANO1 gene polymorphisms (rs7127129 and rs2509153), promoter methylation status, and serum chloride levels in patients with idiopathic pulmonary arterial hypertension (IPAH), congenital heart disease (CHD), and chronic thromboembolic pulmonary hypertension (CTEPH). Methods: A total of 106 IPAH patients, 40 CHD patients, and 30 CTEPH patients, together with 125 healthy controls, were included. The control group had a comparable age distribution, with a balanced sex ratio, whereas females predominated in all three PH groups. Genotyping was performed using TaqMan-based real-time PCR. Promoter methylation was analyzed using bisulfite conversion followed by quantitative real-time PCR. Serum chloride levels were measured using an ion-selective electrode method. Results: No significant association was observed between rs7127129 and rs2509153 polymorphisms and IPAH or CTEPH (*p* > 0.05). However, rs7127129 showed a significant association with CHD (*p* < 0.05). After excluding hypertensive patients, both polymorphisms remained significantly associated with CHD. Serum chloride levels differed significantly among groups (*p* < 0.001), with higher levels observed particularly in the CTEPH and CHD groups compared to controls, while IPAH patients exhibited intermediate but still elevated levels relative to controls. In contrast, promoter methylation levels were significantly lower in all patient groups compared to controls. An inverse relationship between chloride levels and methylation status was observed. Conclusions: ANO1 polymorphisms are not major determinants of IPAH or CTEPH but may contribute to CHD susceptibility. Increased serum chloride levels, together with decreased promoter methylation, suggest a potential mechanistic link between ion channel dysregulation and epigenetic alterations in pulmonary hypertension. Further large-scale and functional studies are warranted.

## 1. Introduction

Pulmonary arterial hypertension (PAH) is a rare but severe pathophysiological disorder characterized by elevated pulmonary arterial pressure and progressive pulmonary vascular remodeling. Its etiology is multifactorial, involving environmental, molecular, and genetic determinants, including gene polymorphisms [1]. According to current clinical classifications and hemodynamic definitions, PAH is characterized by increased pulmonary vascular resistance and mean pulmonary arterial pressure exceeding normal thresholds [2,3]. Among the different subtypes, idiopathic pulmonary arterial hypertension (IPAH) is particularly notable due to its rapid progression, life-threatening nature, and significant impact on patient morbidity and mortality [4].

The pathogenesis of PAH is primarily driven by increased proliferation of pulmonary arterial smooth muscle cells, endothelial dysfunction, and inflammatory cell infiltration, all of which contribute to elevated pulmonary vascular resistance [4,5]. As the disease progresses, sustained pressure overload leads to right ventricular dysfunction and eventual right heart failure. Despite advances in therapeutic strategies, the prognosis of IPAH remains poor, emphasizing the need to better understand its underlying molecular mechanisms. Genetic predisposition plays a critical role in PAH development. Mutations in genes involved in vascular signaling pathways, such as members of the bone morphogenetic protein (BMP) family, have been implicated in idiopathic forms of the disease [6]. However, known genetic mutations do not fully explain the heterogeneity of IPAH, suggesting that additional genetic variations and regulatory mechanisms contribute to disease susceptibility.

Ion channels have recently emerged as key regulators of pulmonary vascular tone and remodeling. In particular, calcium-activated chloride channels (CaCCs) regulate membrane potential, intracellular calcium dynamics, and vascular smooth muscle cell contraction. Anoctamin-1 (ANO1/TMEM16A), a major CaCC, plays a central role in these processes by facilitating chloride (Cl^−^) efflux, membrane depolarization, and secondary calcium influx, ultimately promoting vasoconstriction and vascular remodeling [7,8]. Experimental studies have demonstrated that ANO1 overexpression enhances vascular smooth muscle proliferation and contributes to pathological vascular remodeling, suggesting its involvement in pulmonary hypertension pathogenesis [8,9]. Genetic variations within the ANO1 gene may influence channel expression and function. Among these, rs7127129 and rs2509153 polymorphisms have been previously associated with vascular disorders such as hypertension and are located in regulatory regions that may affect transcriptional activity [7]. These polymorphisms were therefore selected as candidate variants due to their potential functional relevance in modulating ANO1 expression and their previously reported association with vascular phenotypes.

In addition to genetic variation, epigenetic mechanisms such as promoter DNA methylation play a crucial role in regulating gene expression. Alterations in methylation status, particularly promoter hypomethylation, may lead to increased ANO1 expression and enhanced chloride channel activity. Such epigenetic dysregulation may contribute to abnormal vascular responses and disease progression in PAH [10].

Furthermore, chloride homeostasis itself has emerged as an important factor in vascular biology. Changes in intracellular and extracellular Cl^−^ levels influence vascular smooth muscle cell proliferation, apoptosis, and contractility. Increased chloride channel activity has been linked to enhanced vasoconstriction and vascular remodeling, both hallmark features of IPAH [8,11]. Serum chloride levels may therefore serve as an indirect but clinically accessible biomarker reflecting alterations in chloride channel function and vascular physiology.

Taken together, the integration of genetic polymorphisms, epigenetic modifications, and functional biochemical parameters provides a comprehensive framework for understanding PAH pathogenesis. Based on this rationale, the present study aimed to investigate the relationship between ANO1 gene polymorphisms (rs7127129 and rs2509153), serum chloride levels, and promoter methylation status in patients with idiopathic pulmonary arterial hypertension and related pulmonary hypertension subtypes.

## 2. Materials and Methods

### 2.1. Study Population and Clinical Data Collection

**Figure 1:**
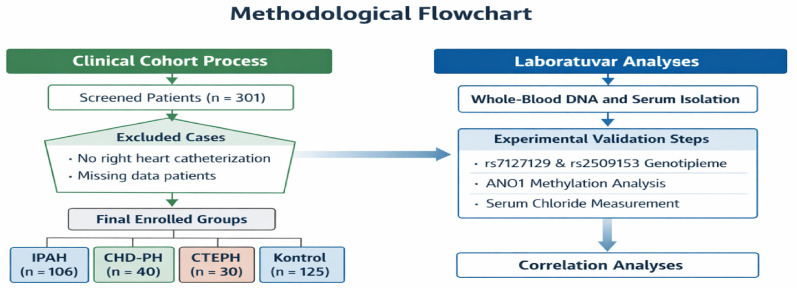
Methodological flowchart of the study. The clinical cohort process is shown on the left: screened patients (*n* = 301), exclusion criteria (no right heart catheterization or missing data), and final enrolled groups [IPAH (*n* = 106), CHD-PH (*n* = 40), CTEPH (*n* = 30), and controls (*n* = 125)]. The laboratory workflow is presented on the right: whole-blood DNA and serum isolation, experimental validation steps [rs7127129 and rs2509153 genotyping, ANO1 promoter methylation analysis, and serum chloride measurement]. All data were integrated for correlation analyses.

This study was conducted in 2022 at the Department of Cardiology, Fırat University Faculty of Medicine Hospital. The study population consisted of 106 patients diagnosed with idiopathic pulmonary arterial hypertension (IPAH), 40 patients with Group 1 pulmonary hypertension associated with congenital heart disease, and 30 patients with chronic thromboembolic pulmonary hypertension (CTEPH). In addition, 125 healthy individuals without any personal or family history of pulmonary hypertension were included as the control group. The control group had a comparable age distribution with a balanced sex ratio, whereas females predominated in all three PH groups. In the congenital heart disease-associated pulmonary hypertension group, the diagnoses of the 40 patients were as follows: atrial septal defect (ASD) in 22 patients, ventricular septal defect (VSD) in 14 patients, and patent ductus arteriosus (PDA) in 4 patients. The mean age and sex distribution were as follows: IPAH group, 58.7 ± 14.2 years (female/male: 84.9%/15.1%); congenital heart disease-associated PH group, 45.3 ± 14.6 years (female/male: 72.5%/27.5%); CTEPH group, 64.5 ± 14.0 years (female/male: 66.7%/33.3%); and control group, 44.9 ± 18.9 years (female/male: 50.4%/49.6%). All participants were informed in detail about the study protocol, and written informed consent was obtained from each individual prior to enrollment. Clinical evaluation included detailed medical history, electrocardiography (ECG), transthoracic echocardiography (ECHO), and peripheral blood sampling. Venous blood samples (3 mL) were collected in EDTA-containing tubes, centrifuged, and stored at −20 °C until further analysis. Echocardiographic assessments were performed using a GE Vivid E9 system (GE Healthcare, Horten, Norway), and all relevant cardiac parameters were systematically recorded and documented. The overall methodological design of the study is illustrated in Figure 1. The flowchart illustrates the sequential process from patient screening and eligibility assessment to final cohort grouping and subsequent laboratory analyses. On the left side, the clinical cohort process includes screened patients, exclusion criteria (absence of right heart catheterization or missing data), and the final enrolled groups (IPAH, CHD-PH, CTEPH, and control). On the right side, the laboratory workflow demonstrates the experimental validation steps, including rs7127129 and rs2509153 genotyping, ANO1 promoter methylation analysis, and serum chloride measurement. All collected data were integrated for correlation analyses.

### 2.2. Molecular Genetic Analyses

#### 2.2.1. DNA Isolation and Quality Assessment

Genomic DNA was extracted from peripheral blood samples using a commercially available Wizard^®^ Genomic DNA Purification Kit (Cat. No. A1125, Promega Corporation, Madison, WI, USA), following the manufacturer’s protocol. Blood samples were collected in EDTA-coated tubes and stored at −20 °C until processing. Prior to extraction, samples were thawed at room temperature. Following isolation, DNA samples were subjected to an additional washing step with 300 µL of 70% ethanol, air-dried, and resuspended in 200 µL of nuclease-free distilled water (ddH_2_O). The purified DNA samples were stored at −20 °C until use in downstream genotyping and methylation analyses. DNA concentration and purity were determined using a MaestroNano spectrophotometer (Maestrogen Inc., Hsinchu, Taiwan). The absorbance ratio at 260/280 nm (A260/A280) was used to assess DNA purity, with values around 1.8 considered acceptable for high-quality DNA. Samples with suboptimal purity were reprocessed either by protein precipitation or re-isolation from whole blood.

#### 2.2.2. TaqMan-Based Genotyping by Real-Time PCR

Genotyping of the ANO1 gene polymorphisms rs7127129 and rs2509153 was performed using TaqMan allelic discrimination assays on an ABI 7500 Fast Real-Time PCR System (Applied Biosystems, Foster City, CA, USA). Genomic DNA samples were diluted to a working concentration of 1–10 ng/µL prior to amplification. PCR reactions were prepared on ice in 96-well plates with a final reaction volume containing 2.5 µL of genomic DNA, 5 µL of TaqMan Universal PCR Master Mix, 0.25 µL of TaqMan SNP Genotyping Assay, and 1.0 µL of nuclease-free water. Plates were sealed with optical adhesive film and centrifuged briefly before amplification. Thermal cycling conditions consisted of an initial denaturation step at 95 °C for 10 min, followed by 45 cycles of denaturation at 95 °C for 15 s and annealing/extension at 60 °C for 1 min. Allelic discrimination analysis was carried out using the Real-time PCR data were analyzed using 7500 Fast Real-Time PCR Software v2.3 (Applied Biosystems, Foster City, CA, USA), which classified samples into homozygous wild-type, heterozygous, or homozygous variant genotypes based on fluorescence signal detection. All procedures were conducted in accordance with the manufacturer’s instructions to ensure high analytical sensitivity and specificity (Table 1). 

#### 2.2.3. Analysis of ANO1 Promoter Methylation by Bisulfite Conversion and Quantitative Real-Time PCR (qRT-PCR)

Genomic DNA samples obtained from patients and controls were subjected to bisulfite conversion using the Methyledge™ Bisulfite Conversion System (Catalog No.: N1301, Promega, Madison, WI, USA) according to the manufacturer’s instructions. The methylation assay targeted a CpG-rich region within the ANO1 locus previously described by Shin et al. [12]. The analyzed region corresponds to the ANO1 CpG island located on chromosome 11 (NCBI Reference Sequence NC_000011: 70,078,169–70,079,120), spanning 952 bp and containing 128 CpG sites. Following bisulfite conversion, methylation-specific and unmethylation-specific primer pairs were used to amplify CpG-containing sequences within this regulatory region. Quantitative methylation analysis was subsequently performed using SYBR Green-based real-time PCR on an ABI 7500 Fast Real-Time PCR System (Applied Biosystems, Foster City, CA, USA). The primer sequences used for methylated and unmethylated DNA amplification are presented in Table 2 and were adopted from the study of Shin et al. (2024), in which this CpG island was identified as an important epigenetic regulatory region associated with ANO1 transcriptional activity [12,13]. For the detection of methylated sequences, LM/RM primers specific to ANO1 and the reference gene β-actin were used, whereas LUM/RUM primers were employed for the amplification of unmethylated sequences. Each sample was analyzed in triplicate, and two separate reactions (methylated and unmethylated) were performed. PCR reactions were prepared in 96-well plates with a total reaction volume of 5 µL per well, consisting of 1 µL bisulfite-converted DNA, 2.5 µL Enturbo™ SYBR Green PCR Supermix (Cat. No. BMB-Q001-EQ00, ELK Biotechnology Co., Ltd., Sugar Land, TX, USA), 0.5 µL of each forward and reverse primer (LM/RM or LUM/RUM), and 0.5 µL nuclease-free water. Plates were sealed, briefly centrifuged, and loaded into the ABI 7500 Fast Real-Time PCR system. Thermal cycling conditions included an initial denaturation step at 95 °C for 30 s, followed by 40 cycles of denaturation at 95 °C for 5 s, annealing at 60 °C for 30 s, and extension at 72 °C for 30 s. Following amplification, quantitative analysis of methylation levels was performed using the 7500 Fast Real-Time PCR Software v2.3 (Applied Biosystems, Foster City, CA, USA). The methylation index (MI) and weighted methylation index were determined based on the ratio between the methylation level of the target gene (ANO1) and the internal reference gene (β-actin), obtained via SYBR analysis [14]. Cycle threshold (Ct) values were automatically converted to relative expression levels using the 2^−ΔΔCt^ method. For statistical analysis, the ratio of methylated to unmethylated DNA (2^−ΔΔCt^ methylated/2^−ΔΔCt^ unmethylated) was calculated. The MI was calculated using the following formula: MI (%) = (methylation level of target gene/methylation level of β-actin) × 100. This ratio was used as a quantitative measure representing the relative methylation level in each sample.

#### 2.2.4. Cl^−^ Measurement

The Cl^−^ level was measured as mmol/L in the serum sample automatically diluted with an ion-selective electrode on the Roche Cobas c 501 autoanalyzer with original kits (Lot: 76939201). Ion-selective electrodes (ISEs) utilize the intrinsic properties of certain membrane materials to develop an electric potential for the purpose of measuring ions in solution. The electrode has a selective membrane that is in contact with both the test solution and an internal filling solution. The internal filling solution contains a fixed concentration of test ions. Due to the special structure of the membrane, the test ions will be closely associated with the membrane on both sides. The electric potential value of the membrane is determined by the difference in the concentration of the test ion in the test solution and the internal filling solution.

#### 2.2.5. Statistical Analysis

All statistical analyses were performed using IBM SPSS Statistics version 26.0 (IBM Corp., Armonk, NY, USA). Data were analyzed with a focus on comparisons between disease subgroups, overall patient and control groups, and allele/genotype distributions. The normality of data distribution was assessed using the Shapiro–Wilk test, and homogeneity of variances was evaluated using Levene’s test. Comparisons between two groups were performed using the independent-samples *t*-test or Mann–Whitney U test, as appropriate, while comparisons among multiple groups were conducted using one-way ANOVA or the Kruskal–Wallis test, followed by post hoc analyses where necessary. Genotype and allele frequencies of rs7127129 and rs2509153 were compared between groups using the chi-square (χ^2^) test or Fisher’s exact test when appropriate. Hardy–Weinberg equilibrium (HWE) was assessed for each polymorphism in the control group. Associations between genetic variants and clinical parameters were evaluated using correlation analyses and logistic regression models, where applicable. Odds ratios (ORs) and 95% confidence intervals (CIs) were calculated to estimate the strength of associations. A two-tailed *p*-value of <0.05 was considered statistically significant.

## 3. Results

### 3.1. Genotype Distribution Results

**Figure 2:**
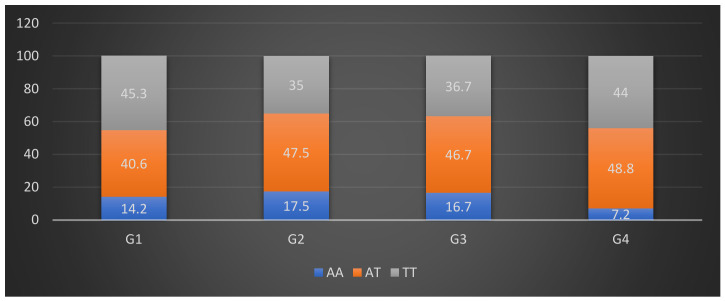
Association between rs2509153 allele combinations and disease groups. Group definitions: G1: idiopathic pulmonary arterial hypertension (IPAH); G2: congenital heart disease (CHD); G3: chronic thromboembolic pulmonary hypertension (CTEPH); G4: control group.

**Figure 3:**
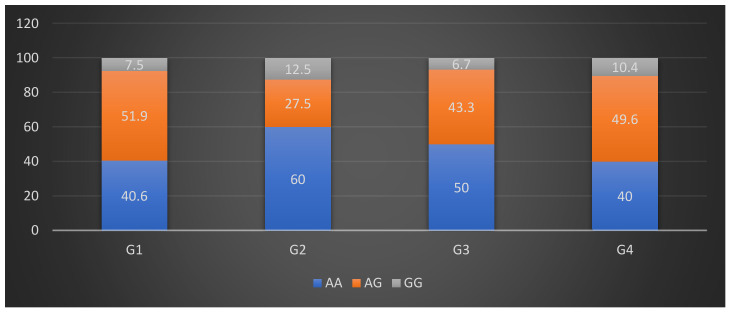
Association between rs7127129 allele combinations and disease groups. Group definitions: G1: idiopathic pulmonary arterial hypertension (IPAH); G2: congenital heart disease (CHD); G3: chronic thromboembolic pulmonary hypertension (CTEPH); G4: control group.

No significant association was observed between the ANO1 gene polymorphisms rs7127129 and rs2509153 and the etiology of idiopathic pulmonary arterial hypertension (IPAH) or chronic thromboembolic pulmonary hypertension (CTEPH) (*p* > 0.05). While the rs2509153 polymorphism was not associated with any disease group, a statistically significant association was identified between the rs7127129 polymorphism and congenital heart disease (CHD) (*p* < 0.05). To further evaluate the relationship between genotype distributions and disease groups, chi-square (χ^2^) analyses were performed for rs2509153 allele combinations. The results demonstrated no statistically significant differences in genotype distributions among IPAH (G1), CHD (G2), CTEPH (G3), total patient group (G5), and control group (G4) (all *p* > 0.05). Similarly, allele frequency analysis revealed no significant differences between groups. The frequency of the A allele ranged between 0.31 and 0.41, while the T allele ranged between 0.59 and 0.69 across groups. Chi-square analysis of allele distributions confirmed the absence of statistically significant associations (all *p* > 0.05). Odds ratio (OR) analyses also indicated no significant association between rs2509153 genotypes or alleles and disease risk. OR values across comparisons ranged from 0.66 to 3.05, with all 95% confidence intervals crossing unity, further supporting the lack of association (Table 3, Figure 2 and Figure 3).

It was observed that the statistically significant association initially detected between the rs7127129 polymorphism and congenital heart disease (CHD) persisted in subgroup analyses performed after excluding cases diagnosed with hypertension (*p* < 0.05). This finding suggests that the observed association is independent of the presence of hypertension (Table 4).

### 3.2. ANO1 Promoter Methylation and Serum Chloride Levels

Serum chloride levels and methylation ratio (methylation index) values were compared across study groups using the Kruskal–Wallis H test. The results demonstrated statistically significant differences among groups for both parameters. For serum chloride levels, a significant difference was observed across groups (χ^2^ = 143.15, *p* < 0.001). The mean chloride levels were 106.00 ± 10.51 in the IPAH group (G1), 115.44 ± 15.32 in the congenital heart disease group (G2), 129.57 ± 18.26 in the CTEPH group (G3), and 96.06 ± 6.41 in the control group (G4). Post hoc pairwise comparisons revealed that chloride levels in G1 were significantly lower than those in G2 (*p* = 0.001) and G3 (*p* < 0.001). Additionally, chloride levels in the control group (G4) were significantly lower than those in G1 (*p* < 0.001) and G2 (*p* < 0.001). Furthermore, G2 patients exhibited significantly lower chloride levels compared to G3 (*p* = 0.001). To evaluate the potential confounding effect of medication use on serum chloride levels, additional analyses were performed in the patient cohort. No significant associations were observed between serum chloride concentrations and the use of beta-blockers (*p* = 0.609), calcium channel blockers (*p* = 0.813), diuretics (*p* = 0.220), antiplatelet agents (*p* = 0.310), anticoagulants (*p* = 0.975), single PH-specific therapy (*p* = 0.856), dual PH-specific therapy (*p* = 0.754), or triple PH-specific therapy (*p* = 0.516). These findings suggest that the elevated chloride levels observed in the patient groups were not significantly influenced by the recorded medication profiles.

Analysis of methylation ratio values also revealed a statistically significant difference among groups (χ^2^ = 32.83, *p* < 0.001). The mean methylation values were 1.96 ± 2.45 (G1), 1.81 ± 2.28 (G2), 1.81 ± 2.79 (G3), and 5.15 ± 9.95 (G4). Post hoc analysis indicated that the control group (G4) had significantly higher methylation levels compared to all patient groups, including G1 (*p* < 0.001), G2 (*p* < 0.001), and G3 (*p* < 0.001) (Table 5).

## 4. Discussion

Our study was designed based on the critical role of the calcium-activated chloride channel Anoctamin-1 (ANO1/TMEM16A) in the regulation of vascular tone. In this context, we aimed to investigate the relationship between ANO1 gene polymorphisms (rs7127129 and rs2509153), promoter methylation status, and serum chloride levels in patients with idiopathic pulmonary arterial hypertension (IPAH), congenital heart disease (CHD), and chronic thromboembolic pulmonary hypertension (CTEPH).

Although numerous studies have investigated the etiology and treatment of idiopathic pulmonary arterial hypertension (IPAH), its underlying mechanisms remain incompletely understood. Increasing evidence suggests that genetic polymorphisms may contribute to disease susceptibility, and studies focusing on this aspect have increased considerably in recent years [15,16]. In particular, genes involved in vascular remodeling, ion channel regulation, and inflammatory signaling pathways have been implicated in pulmonary vascular dysfunction [2,3,4].

Recent studies have highlighted a potential association between ANO1 gene variants and hypertension. Jin and Jung demonstrated that ANO1 polymorphisms may exert sex-specific effects, particularly increasing hypertension risk in males [7]. These findings support the hypothesis that genetic variation interacts with environmental and hormonal factors to influence vascular tone and disease susceptibility. In line with this, calcium-activated chloride channels such as TMEM16A (ANO1) play a crucial role in vascular smooth muscle contraction and membrane depolarization, thereby regulating arterial tone and contributing to hypertensive processes [17,18].

In another study investigating ANO1 polymorphisms in pulmonary hypertension, reduced gene expression was suggested to increase disease susceptibility; however, no statistically significant association was found between rs2509153 and disease risk [17]. Similarly, experimental studies have demonstrated that TMEM16A contributes to Ca^2+^-dependent chloride currents that promote vascular smooth muscle depolarization and vasoconstriction, which are key features of pulmonary hypertension [9,18].

In the present study, no significant association was observed between rs2509153 and pulmonary hypertension subtypes. However, a statistically significant difference was identified between the control group and the congenital heart disease (CHD) group for rs7127129. This finding may be explained by the role of ANO1 in myocardial remodeling and fibrosis. Previous studies have demonstrated that reduced ANO1 expression contributes to increased cardiac pressure and fibrosis, whereas increased expression suppresses the TGF-β/Smad3 signaling pathway and attenuates fibrotic responses [19,20]. Furthermore, ANO1 overexpression in vascular smooth muscle cells has been shown to increase vascular tone, while its inhibition leads to reduced blood pressure [9,17].

Supporting these findings, ANO1-mediated chloride currents have been shown to play a central role in vascular smooth muscle excitability and contractility. The depolarizing effect induced by chloride efflux contributes to increased intracellular calcium levels, thereby enhancing vasoconstriction [9,19,20,21]. This mechanism is particularly relevant in pulmonary circulation, where small changes in vascular tone can lead to significant increases in pulmonary arterial pressure [2,3].

In our study, no statistically significant association was found between rs2509153 or rs7127129 polymorphisms and the overall patient group (IPAH, CHD, and CTEPH combined). However, subgroup analyses revealed significant differences between CHD patients and controls for both polymorphisms after excluding hypertensive patients. This suggests that these variants may have disease-specific effects rather than a generalized role in pulmonary hypertension, consistent with previous reports highlighting context-dependent genetic effects [16,22]. A search of publicly available GWAS repositories, including OpenGWAS and FinnGen, did not identify datasets reporting rs7127129 or rs2509153 in association with pulmonary hypertension phenotypes. Therefore, independent GWAS-based validation of these variants is currently unavailable. A summary of the external GWAS screening results is provided in Table A1. These results support the conclusion that these ANO1 variants are unlikely to be major genetic determinants of IPAH or CTEPH. The rs7127129 association observed in the CHD subgroup may represent a subtype-specific effect and warrants further investigation in larger cohorts.

Experimental studies further support the involvement of ANO1 in cardiac remodeling. Tian et al. demonstrated that ANO1 expression increases following myocardial infarction and promotes cardiac fibroblast proliferation, whereas its inhibition suppresses fibrotic activity [19]. Similarly, Gao et al. showed that ANO1 regulates fibrosis through the TGF-β/Smad3 pathway [20]. These findings suggest that ANO1 may play a dual role in cardiovascular diseases by modulating both vascular tone and fibrotic remodeling.

Other genetic studies have identified associations between cardiovascular diseases and polymorphisms in ion channel-related genes such as rs3737964 and rs10927887, further emphasizing the multifactorial genetic basis of cardiovascular pathology [23,24]. In addition, pulmonary arterial hypertension has been linked to rare pathogenic variants in genes such as BMPR2, highlighting the importance of genetic predisposition and hereditary factors in disease development [6,16,22].

In addition to genetic findings, our study demonstrated a significant association between serum chloride levels and disease groups. Given the role of ANO1 as a chloride channel, these findings support the hypothesis that ionic imbalance contributes to disease pathophysiology. Alterations in chloride homeostasis may influence membrane potential, vascular smooth muscle excitability, and endothelial function, thereby contributing to abnormal vascular tone and remodeling [9,21]. Serum chloride levels were significantly elevated in all pulmonary hypertension groups compared with controls. Given the role of ANO1 as a calcium-activated chloride channel, this finding may suggest an association between altered chloride homeostasis and pulmonary vascular disease (Duran et al., 2010; Yuan et al., 2023) [11,17]. However, serum chloride concentrations may also be influenced by factors such as medication use, renal function, and acid–base balance [25]. In additional analyses, no significant associations were observed between serum chloride levels and the use of diuretics or other cardiovascular medications (all *p* > 0.05). Therefore, the observed hyperchloremia should be interpreted as an association with pulmonary hypertension rather than direct evidence of ANO1-mediated vascular remodeling.

Recent studies have demonstrated that endothelial TMEM16A channels are activated by TRPV4-mediated Ca^2+^ influx, leading to arterial relaxation. These findings contrast with our observation of vasoconstrictive effects in smooth muscle cells, suggesting that TMEM16A may exert cell-type-dependent dual roles in vascular tone regulation. Recent studies have demonstrated that endothelial TMEM16A channels are activated by TRPV4-mediated Ca^2+^ influx, leading to arterial relaxation (Mata-Daboin et al. [26]). These findings contrast with our observation of vasoconstrictive effects in smooth muscle cells, suggesting that TMEM16A may exert cell-type-dependent dual roles in vascular tone regulation. In another recent study, genetic deletion or pharmacological inhibition of TMEM16A channels in vascular smooth muscle was shown to reduce arterial tone and systemic blood pressure, while inhibition in cortical pericytes protected against ischemic injury and improved microvascular flow. In endothelial cells, TMEM16A was reported to regulate vascular tone through modulation of cell proliferation and transmission of hyperpolarization to smooth muscle cells. Genetic evidence further implicates TMEM16A in systemic and pulmonary hypertension, stroke, and Moyamoya disease. These findings align with our observations of TMEM16A polymorphisms and promoter methylation in pulmonary hypertension subgroups, underscoring the channel’s multifaceted and cell-type-dependent roles in vascular pathophysiology [27].

Furthermore, methylation analysis revealed that the control group exhibited significantly higher methylation levels compared to all patient groups. Epigenetic mechanisms, particularly DNA methylation, are known to regulate gene expression dynamically and may influence ANO1 transcriptional activity [12,15]. The observed inverse relationship between chloride levels and methylation ratios suggests a potential interaction between ion channel function and epigenetic regulation. However, an important limitation of the present study should be acknowledged. Methylation analyses were performed using genomic DNA isolated from peripheral blood leukocytes rather than pulmonary vascular tissue. Therefore, the detected methylation changes cannot be assumed to directly reflect epigenetic regulation within pulmonary arterial smooth muscle cells or endothelial cells, which are the principal cellular mediators of pulmonary vascular remodeling. Consequently, the observed ANO1 promoter hypomethylation should be interpreted as a systemic peripheral blood-associated epigenetic signature rather than direct evidence of local vascular pathogenic mechanisms. Future studies incorporating pulmonary vascular tissue, isolated endothelial cells, or pulmonary artery smooth muscle cells are required to establish tissue-specific epigenetic regulation of ANO1 in pulmonary hypertension.

Taken together, our findings suggest that ANO1 promoter hypomethylation and elevated serum chloride levels are associated with pulmonary hypertension. However, because methylation analyses were performed using peripheral blood-derived DNA, these observations should be considered potential systemic biomarkers of disease status rather than direct evidence of pulmonary vascular remodeling. Further tissue-specific and functional studies are needed to clarify the mechanistic role of ANO1 epigenetic regulation in pulmonary hypertension. However, this study has several limitations, including a relatively limited sample size and the lack of functional validation experiments. Future studies should include larger cohorts and integrate genomic, epigenomic, and functional analyses to better elucidate the role of ANO1 in pulmonary hypertension [22,28].

## Figures and Tables

**Table 1:** Reference numbers and sequences of polymorphisms in the ANO1 gene are presented.

Test Name/Reference Number	DNA Sequences	Polymorphism Type
rs7127129	AAAAAAAAAAAAGTAGTAGCAGGAA**[A/G]**CATTCACGAGGCACCTAGAATGTTC	Intronic, A/G, Conversion
rs2509153	CTGGAATTTGAGGAGTCTCGGGCCT**[A/T]**ATCCCAGCTCATCAGCACAAATAAA	Intronic, A/T, Transversion Substitution

For both polymorphisms, the A allele was labeled with VIC, while the G and T alleles were labeled with FAM.

**Table 2:** Primer sequences used for bisulfite conversion-based methylation analysis of the ANO1 gene [14,15].

Primer Name	Base Sequences 5′-3′
ANO1 LM	5′-TTTTAAGGTAAAGGCGGGTC-3′
ANO1 RM	5′-CTCGATACGAAAAACGCCTA-3′
Ano1 LUM	5′-TATTTTTAAGGTAAAGGTGGGTT-3′
Ano1 RUM	5′-CTCAATACAAAAAACACCTAAAC-3′
ActB LM	5′-TGGTGATGGAGGAGGTTTAGTAAGT-3′
ActB RM	5′-AACCAATAAAACCTACTCCTCCCTTAA-3′
ActB LUM	5′-TGGTGATGGAGGAGGCTCAGCAAGT-3′
ActB RUM	5′-AGCCAATGGGACCTGCTCCTCCCTTGA-3

LM: left methylated, LUM: left unmethylated, RM: right methylated, RUM: right unmethylated.

**Table 3:** Association between rs2509153 and rs7127129 genotype and allele distributions across study groups.

rs2509153		Groups				
Genotypes		G1	G2	G3	G5	G4
AA	*n* (%)	15 (%14.2)	7 (%17.5)	5 (%16.7%)	27 (%15.3)	9 (7.2%)
AT	*n* (%)	43 (%40.6)	19 (%47.5)	14 (%46.7)	76 (543.2)	61 (%48.8)
TT	*n* (%)	48 (%45.3)	14 (%35.0)	11 (%36.7)	73 (%41.5)	55 (44%)
Ki kare *p*-value OR		3.552 0.169—*p* > 0.05 1.91 (0.76–4.75)	3.913 0.141—*p* > 0.05 3.05 (0.96–9.63)	2.729 0.256—*p* > 0.05 2.26 (0.98–5.19)	4.66 0.97—*p* > 0.05 2.77 (0.78–9.89)	
Alleles		G1	G2	G3	G5	G4
A		(0.344)	0.41	0.4	0.36	0.31
T		(0.656)	0.59	0.6	0.64	0.69
Ki kare *p*-value OR		0.42 0.55—*p* > 0.05 0.66 (0.39–1.11)	2.52 0.135—*p* > 0.05 0.658 (0.39–1.1)	1.54 0.224—*p* > 0.05 0.693 (0.38–1.23)	1.83 0.192—*p* > 0.05 0.78 (0.55–1.11)	
Rs7127129		Groups				
Genotypes		G1	G2	G3	G5	G4
AA	*n*%	43 (40.6)	24 (60.0%)	15 (50.0%)	82 (46.6%)	50 (%40.0)
AG	*n*%	55 (%51.9)	11 (21.5%)	13 (43.3%)	79 (44.9%)	62 (%49.6)
GG	*n* %	8 (7.5%)	5 (12.5%)	2 (6.7%)	15 (8.5%)	13 (%10.4)
Ki kare *p*-value OR		0.57 0.74—*p* > 0.05 1.39 (0.53–3.68)	6.17 0.04—*p* < 0.05 1.24 (1.19–3.90)	1.12 0.57—*p* > 0.05 1.95 (0.39–9.62)	1.34 0.51—*p* > 0.05 1.42 (0.62–3.23)	
Alleles		G1	G2	G3	G5	G4
A		0.66	0.73	0.71	0.69	0.64
G		0.34	0.27	0.29	0.31	0.36
Ki kare *p*-value OR		0.15 0.76—*p* > 0.05 0.92 (0.63–1.36)	2.19 0.17—*p* > 0.05 0.65 (0.37–1.14)	1.02 0.36—*p* > 0.05 0.72 (0.99–1.35)	1.19 0.29—*p* > 0.05 0.82 (0.58–1.16)	

Group definitions: G1: idiopathic pulmonary arterial hypertension (IPAH); G2: congenital heart disease (CHD); G3: chronic thromboembolic pulmonary hypertension (CTEPH); G4: control group; G5: total patient group.

**Table 4:** Group analyses for rs7127129 after exclusion of patients with systemic hypertension.

rs7127129	Groups				
Genotypes	G1	G2	G3	G5	G4
AA	21 (%39.6)	19 (%67.8)	6 (%35.3)	46	50 (%40.0)
AG	26 (%49.1)	8 (%28.6)	9 (%52.9)	43	62 (%49.6)
GG	6 (%11.3)	1 (%3.6)	2 (%11.8)	9	13 (%10.4)
Chi-square *p*-value OR	1.165 0.558—*p* > 0.05 1.00 (0.50–1.98)	7.313 0.026—*p* < 0.05 2.94 (1.19–7.28)	0.144 0.931—*p* > 0.05 0.82 (0.27–2.47)	1.079 0.583—*p* > 0.05 0.82 (0.27–2.47)	6.347 0.026—*p* < 0.05
Alel	G1	G2	G3	G5	G4
A	0.64	0.82 ile	0.617		0.648
G	0.36	0.18	0.383		0.352
Chi-square *p*-value OR	0.01 0.99—*p* > 0.05 1.02 (0.64–1.65)	6.32 0.016, *p* < 0.05 0.40 (0.19–0.83)	0.12 0.848—*p* > 0.05 1.13 (0.54–2.38)	0.82 ile 0.418—*p* > 0.05 0.83 (0.58–1.23)	

Group definitions: G1: idiopathic pulmonary arterial hypertension (IPAH); G2: congenital heart disease (CHD); G3: chronic thromboembolic pulmonary hypertension (CTEPH); G4: control group; G5: total patient group.

**Table 5:** Results of the association between serum chloride levels and methylation ratio values across disease groups.

Cl Values	Groups			
	G1	G2	G3	G4
***n***	106	40	30	125
**Mean ± SD**	106.00 ± 10.51	115.44 ± 15.32	129.57 ± 18.26	96.06 ± 6.41
**Analysis/Difference Results**	X^2^ = 143.15 *p* = 0.000/G^1^ < G^2^- G^1^ < G^3^- G^4^ < G^1^- G^4^ < G^2^- G^2^ < G^3^			
**Methylation values**	**Groups**			
	**G1**	**G2**	**G3**	**G4**
***n***	106	40	30	125
**Mean ± SD**	1.96 ± 2.45	1.81 ± 2.28	1.81 ± 2.79	5.15 ± 9.95
**Analysis/Difference Results**	X^2^ = 32.83 *p* = 0.000/G^1^ < G^4^- G^2^ < G^4^- G^3^ < G^4^			

Abbreviations used in this study are as follows: G1: idiopathic pulmonary arterial hypertension (IPAH); G2: congenital heart disease (CHD); G3: chronic thromboembolic pulmonary hypertension (CTEPH); and G4: control group. Descriptive statistics are presented as mean (arithmetic mean), standard deviation (SD), Statistical comparisons between groups were performed using the Kruskal–Wallis H test (χ^2^).

## Data Availability

The data supporting the findings of this study are available from the corresponding author upon reasonable request.

## Appendix A

**Table A1:** External GWAS screening results for ANO1 variants in FinnGen R12 and OpenGWAS databases.

Variant	Gene	Database	Pulmonary Hypertension-Related Phenotype	Result
rs7127129	ANO1	FinnGen R12	Pulmonary hypertension	No phenotype record identified
rs7127129	ANO1	FinnGen R12	Overall phenotype screening	No genome-wide significant association observed (all *p* > 5 × 10^−8^)
rs2509153	ANO1	FinnGen R12	Pulmonary hypertension	No phenotype record identified
rs2509153	ANO1	FinnGen R12	Overall phenotype screening	No genome-wide significant association observed (all *p* > 5 × 10^−8^)
rs7127129	ANO1	OpenGWAS	Pulmonary hypertension	No eligible record identified
rs2509153	ANO1	OpenGWAS	Pulmonary hypertension	No eligible record identified
